# Insights into the phytochemical profiling, antidiabetic and antioxidant potentials of *Lepionurus sylvestris* Blume extract in fructose/streptozotocin-induced diabetic rats

**DOI:** 10.3389/fphar.2024.1424346

**Published:** 2024-07-12

**Authors:** Xianzhu Pan, Opeyemi Joshua Olatunji, Abdul Basit, Sasikarn Sripetthong, Sirinporn Nalinbenjapun, Chitchamai Ovatlarnporn

**Affiliations:** ^1^ Department of Pathology and Pathophysiology, Anhui Medical College, Hefei, China; ^2^ African Genome Center, University Mohammed VI Polytechnic, Ben Guerir, Morocco; ^3^ Department of Pharmaceutical Chemistry, Faculty of Pharmaceutical Sciences, Prince of Songkla University, Songkhla, Thailand; ^4^ Drug Delivery System Excellent Center, Faculty of Pharmaceutical Sciences, Prince of Songkla University, Songkhla, Thailand

**Keywords:** diabetes mellitus, Lepionurus sylvestris Blume, glucose intolerance, oxidative stress, inflammatory cytokines, Antioxidants

## Abstract

In this study, the antidiabetic activities of Lepionurus sylvestris Blume extract (LSB) in rats was investigated. The *in vitro* antidiabetic properties of LSB was evaluated using α-amylase, α-glucosidase and DPP-IV inhibitory assays, while the antioxidant assay was analysed using DPPH, ABTS and FRAP assays. Type 2 diabetes was with high-fructose/streptozotocin, and the diabetic animals were treated with LSB for 5 weeks. At the end of the experiment, the effects of LSB were evaluated via insulin level, lipid profile and hepatorenal function biomarkers. The level of oxido-inflammatory parameters, histopathology and insulin immunohistochemical staining in the pancreas was evaluated. Diabetic rats manifested significant increases in the blood glucose level, food/water intake, lipid profiles, hepatorenal function biomarkers, as well as a marked decreases in the body weight and serum insulin levels. Histopathological and insulin immunohistochemical examination also revealed decreased pancreatic beta cells and insulin positive cells, respectively. These alterations were associated with significant increases in malondialdehyde, TNF-α and IL-1β, in addition to significant declines in GSH, SOD and CAT activities. LSB significantly reduced blood glucose level, glucose intolerance, serum lipids, restored altered hepatorenal and pancreatic functions in the treated diabetic rats. Further, LSB showed antioxidant and anti-inflammatory activities by reducing malondialdehyde, TNF-α, IL-1β, and increasing antioxidant enzymes activities in the pancreatic tissues. A total of 77 secondary metabolites were tentatively identified in the UPLC-Q-TOF-MS analysis of LSB. Overall, these findings provides insight into the potentials of LSB as an antidiabetic agent which may be associated to the plethora bioactive compounds in the plant.

## Graphical Abstract

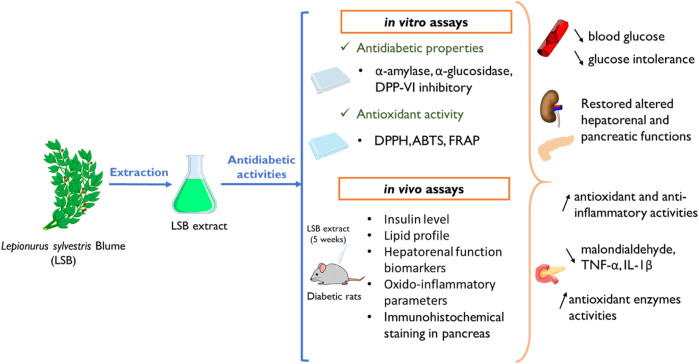

## 1 Introduction

Noncommunicable diseases (NCDs) accounts for 74% of all yearly global mortality and these deaths are attributed to four major diseases; cardiovascular diseases, cancers, chronic respiratory diseases and diabetes (WHO, 2023). Diabetes mellitus is a multiracial NCD that affects over half a billion people globally. The occurrence of diabetes is more severe in low and middle income countries, three out of every four diabetic adults live in these countries (International Diabetes Federation, 2021). Type 2 diabetes (T2D) is the most common type of diabetes and it is depicted by high blood glucose level (or hyperglycemia) resulting from impaired insulin action/secretion or both (Gao et al., 2023). Besides, diabetes leads to several life threatening micro and macro vascular complications, including cardiovascular diseases, nephropathy, neuropathy and retinopathy (Forbes and Cooper, 2013). Furthermore, diabetic patients have increased susceptibility to several cancers including bladder, breast, liver, pancreatic, colorectal and prostate cancers (Giovannucci et al., 2010; Wang et al., 2020). Hyperglycemia can also initiate other chronic diseases such as Alzheimer, myocardial injuries and infections (Koromantzos et al., 2011; Pang et al., 2021). As such, the development of therapeutics for the prevention and treatment of diabetes has become paramount so as to avoid these life threatening diabetic comorbidities. Proper glycaemic control remains the major clinical strategy for managing diabetes and presently the most successful approach for the treatment of diabetes involves the use of hypoglycemic agents (biguanides, sulfonylureas, meglitinide, thiazolidinedione, dipeptidyl peptidase four inhibitors, sodium-glucose cotransporter and alpha-glucosidase inhibitors) together with lifestyle changes. However, prolonged use of these antidiabetic agents have shown several side effects, efficacy issues, cost issues, hypoglycaemia risk and inability to delay diabetic comorbidities (Olokoba et al., 2012; Chaudhury et al., 2017; Ekperikpe et al., 2019; Makinde et al., 2020).

Medicinal plants has successfully been positioned as a potent alternative for the treatment of several chronic diseases. Several developing nations have incorporated the use of herbal medicine in their primary healthcare (Kumar et al., 2015; Makinde et al., 2020). The use of phyto-therapeutics in the management of diabetes have gain enormous attention due to their low toxicity, availability, perceived efficacy and multi-constituents nature of the extracts from these plants, thus affording them multiple mechanisms of antidiabetic action (Ríos et al., 2015; Tang et al., 2017). *Lepionurus sylvestris* Blume (also known as makmok in Thailand) is a plant belonging to the family Opiliaceae. The plant is traditionally used in Thailand, Malaysia and India for treating diabetes, strengthening the body, promoting blood flow and for treating cervical problems. The leaves of the plant is also consumed along with bean seeds to prevent faecal odour (Maneenoon et al., 2015; Thangliankhup et al., 2023). The biological activities or the phytochemical composition of *Lepionurus sylvestris* is largely unexplored. In fact, only two studies has explored the phytochemical and biological properties of the plant (Meerungrueang and Panichayupakaranant, 2014; Phoopha et al., 2023). Hence, the present study investigated the antidiabetic potentials of *L. sylvestris* ethanolic extract in fructose and streptozotocin induced T2D rats.

## 2 Materials and methods

### 2.1 Sample collection, preparation and extraction

*L. sylvestris* was collected from Phatthalung Province, Thailand in November 2021. The plant specimen was identified at the Department of Pharmaceutical Chemistry, Faculty of Pharmaceutical Sciences, Prince of Songkla University, Hat Yai, where the voucher specimen (specimen number 56525671) was deposited. The leaves of the plant were thoroughly washed under running tap water and oven dried at 55°C. The dried leaves were fine powdered with a herb grinder. Thereafter, the ethanolic extract was prepared by macerating 150 g of the powdered sample in 1.5 L of 70% ethanol. The mixture was placed on a shaker at room temperature for 24 h. The procedure was repeated thrice and the extracts were pulled together, filtered and dried to a third of the original volume under reduced pressure. The remaining solution was kept at 4°C overnight and the clear upper hydrophobic layer was carefully decanted, filtered and lyophilized to obtain a brownish hygroscopic powder (LSB), which was stored at −20°C until further use.

### 2.2 *In vitro* antioxidant assay

#### 2.2.1 DPPH assay

The DPPH assay was performed based on a previous protocol (Kamonwannasit et al., 2013). Briefly, the DPPH working solution was prepared by diluting the DPPH stock solution (50 µM) in methanol to give an absorption of 1 ± 0.02 at 515 nm. Thereafter, 30 µL of LSB extract was added to 170 µL of DPPH working solution and the absorbance readings at 515 nm were taken at room temperature 30 min after the initial mixing. The percentage inhibition of DPPH of the test sample was calculated using the formula below:

$$\%\text{ DPPH inhibition}=\left[\left.\left(\text{Acontrol}-\text{Asample}\right)/\text{Acontrol}\right)\right]\times 100$$

Where: control = absorbance of control (no sample)

As = absorbance of sample solution.

#### 2.2.2 ABTS assay

The ABST assay was performed using a modified procedure of Aadesariya et al. (2017). The ABTS stock solution was prepared through the reaction of 7 mM of ABTS solution and 2.45 mM of potassium persulphate (1:1 v/v). The working solution of ABTS+∙ was obtained by diluting the stock solution in ethanol to give an absorption of 0.70 ± 0.02 at 734 nm. Thereafter, 30 µL of LSB extract was added to 195 µL of ABTS+∙ solution and the absorbance readings at 734 nm were taken at room temperature 30 min after initial mixing. The percentage inhibition of ABTS+∙ of the test samples were calculated by the following formula below:

$$\%\text{ ABTS inhibition}=\left[\left.\left(\text{Acontrol}-\text{Asample}\right)/\text{Acontrol}\right)\right]\times 100$$

Where: Acontrol = absorbance of control (no sample)

Asample = absorbance of sample solution.

#### 2.2.3 FRAP assay

The FRAP assay was conducted in accordance with a previously reported method of Kamonwannasit et al. (2013). The FRAP reagent was prepared by reacting 10 mM TPTZ (2,4,6-tripyridyl-striazine, in 40 mM HCl), 20 mM FeCl_3_ and 300 mM acetate buffer (pH 3.6) (1:1:10 v/v) at 37°C. Thereafter, 100 µL of LSB extract was added to 2000 µL of FRAP reagent and the absorbance readings at 593 nm was taken at 37°C. The results were expressed as milligrams quercetin equivalents per gram of dry weight (mg QE/g d. w.) of the extract.

### 2.3 Phytochemical profiling of LSB

#### 2.3.1 Total phenolic (TPC) and flavonoid content (TFC)

The TPC of the extract was evaluated based a previous protocol (Basit et al., 2022). Briefly, 20 mg of LSB was solubilized in 10 mL of ethanol and 25 µL of the extract solution was added to 100 µL of 25% Folin-Ciocalteu reagent. Thereafter, 75 µL of 10% Na_2_CO_3_ was added to the mixture and incubated at room temperature. After 2 h, the absorbance of the solution was determined at 750 nm. The concentration was calculated using gallic acid as a standard and the results were expressed as milligrams gallic acid equivalents (mg GAE/g d. w.) per gram of dry weight of the extract.

The TFC was determined using aluminium chloride colorimetric assay. Standard solutions of quercetin (20–120 μg/mL) was prepared in methanol and 50 µL of the extract was mixed with 10 µL of 10% aluminium chloride solution, 150 µL of methanol and 10 µL of 1 M sodium acetate in a 96 well plate. The resulting solution was thoroughly mixed and incubated for 40 min at room temperature in the dark. The absorbance of the solution was measured at 415 nm with a microplate reader. TFC was expressed as milligrams quercetin equivalents (QE) per gram of plant extract.

#### 2.3.2 UHPLC-Q-TOF-MS profiling of LSB

For UHPLC-Q-TOF-MS profiling, the sample was prepared as follows: LSB was dissolved in 50% MeOH (1 mg/100 µL), then vortexed thoroughly. Next, the solution was centrifuged at 16,000 rpm for 10 min at 25°C. The resulting supernatant was filtered through a 0.22 µm membrane filter and transferred into amber glass vials for analysis.

In this analysis a Dionex Ultimate 300 UHPLC system (Thermo Fisher Scientific Inc., Waltham, MA, USA) coupled to QTOF Impact II (Bruker Daltonics, Bremen, Germany) was employed for tentative identification of the phytoconstituents in LSB. A C18 column (Thermo Fisher Scientific, Sunnyvale, CA, USA) with a particle size of 1.9 μm, 2.1 × 100 mm was used. The Mobile phase was composed of two solvents; A: 0.1% formic acid in aqueous solution and B: 100% acetonitrile. The elution profile used was gradient elution as follow 0%–99% B over 0–18 min, maintained at 99% B for 5 min, followed by 1% B over 23–23.1 min, and a final hold at 1% B for 5 min, resulting in a total run time of 28 min. The flow rate was set at 0.3 mL/min, Mass range of m/z 50–1,200 and Collision energy was 20.0 eV and 10.0 eV for both positive and negative mode. The acquisition of mass spectra was conducted using electrospray ionization (ESI) in both modes of ionization. Nitrogen gas was used for the nebulizer and collision gas. The collision energy and capillary voltage used for the positive mode were 20.0 eV and 3800 V respectively, while 10 eV and 2500 V were used for the negative mode. The dry temperature was 250°C, and the dry gas flow rate was 8.0 L/min. Identification of the compounds was carried out in accordance mass spectral library, and metlin database.

### 2.4 *In vitro* antidiabetic assay

#### 2.4.1 Dipeptidyl peptidase-IV (DPP-IV) inhibitory activity

The DPP-IV inhibitory activity of LSB extract was evaluated according to a previous report of Al-Masri and colleagues (Al-masri et al., 2009) with some modifications. Diprotin A was used as a positive standard and diluted to various concentrations with Tris-HCl buffer (50 mM, pH 7.5). Each sample solution was prepared by diluting the extract with Tris-HCl buffer to a final concentration of 50 μg/mL. Diprotin A solution or the sample solution (40 μL) was transferred to a 96 microplate, followed by the addition of 20 μL of 0.05 U/mL of DPP-IV enzyme. Thereafter, the mixture was pre-incubated for 10 min at 37°C to enhance the binding capacity of the inhibitor. This was followed by the addition of 100 μL of gly-pro-p-nitroanilide (0.2 mM in Tris-HCl) as a substrate. The incubation continued at 37°C for an additional 30 min. The reaction was terminated with the addition of 30 μL of 25% glacial acetic acid. The absorbance of the solution was measured at 405 nm using a microplate reader. The percentage of inhibition was calculated according to the equation below:

$$\%\text{ Inhibition}=\left[\left(\text{Acontrol }-\text{ Asample}\right)\;/\text{ Acontrol}\right]\times 100$$

where Acontrol = absorbance of DPP-IV solution without sample.

Asample = absorbance of DPP-IV reaction with sample.

#### 2.4.2 Alpha glucosidase inhibitory activity

The α-glucosidase inhibitory activity determination was performed using the procedure of Kumar et al. (2013). The stock sample solutions were prepared at 5,000 μg/mL and further diluted with 50 mM phosphate buffer (pH 6.9) to concentrations in the range of 25–5,000 μg/mL 50 μL of the sample solution was mixed with 50 µL of α-glucosidase enzyme (0.57 unit/mL which was dissolved in 50 mM phosphate buffer, pH 6.9), and the mixture was incubated at 37°C for 10 min. Thereafter, 50 µL of the substrate (15 mg of p-nitrophenyl α-D- glucopyranoside dissolved in 10 mL of 50 mM phosphate buffer, pH 6.9) was added to the mixture and further incubated at 37°C for 20 min. The reaction was stopped by adding 50 µL of 1 M Na_2_CO_3_ solution and the absorbance of the reaction mixture was measured at 405 nm. Acarbose was used as a standard at the same concentration of sample. A blank solution was prepared in a similar manner to that of sample using 50 µL of 50 mM phosphate buffer (pH 6.9) instead of the sample solution and used as the negative control. A blank of sample was prepared by mixing all solutions without the enzyme solution. The α-glucosidase inhibitory activity was taken as 50% inhibitory concentration (IC_50_) and was calculated from linear regression between % inhibitions against sample concentrations using the equation below:

$$\%\text{ inhibition}=\left[\left.\text{Anegative}-\left(\text{Asample}-\text{Abs}\right)/\text{Anegative}\right)\right]\times 100$$

Where: Anegative = absorbance of negative solution (no sample)

Asample = absorbance of sample solution.

Abs = absorbance of blank of sample solution.

The results were represented in terms of IC_50_ (µg/mL).

#### 2.4.3 Alpha amylase inhibitory activity

The α-amylase inhibitory assay was evaluated using previously described procedures (Sudha et al., 2011; Chakrabarti et al., 2014). The stocks sample solutions were prepared at 10,000 μg/mL and further diluted with 20 mM phosphate buffer (pH 6.9) to concentration range of 0.5–10,000 μg/mL. Sample solution (20 µL) was mixed with 20 µL of 20 mM phosphate buffer (pH 6.9) and 20 µL of porcine pancreatic α-amylase solution (12.8 units/mL) and incubated at 37°C for 10 min. Thereafter, 20 µL of 1% starch solution was added to the mixture and further incubated at 37°C for 30 min. The reaction was quenched with 20 µL of 1 M HCl and 100 µL of 2.5 mM iodine test solution was added. Acarbose was used as a standard and was prepared at the same sample concentration. The absorbance was measured with a microplate reader at 630 nm. The α-amylase inhibitory activity was expressed as % inhibition using the equation stated below:

$$\%\text{ inhibition}=\left[1-\left({\text{Ab}}_{2}-{\text{Ab}}_{1}\right)/\left({\text{Ab}}_{4}-{\text{Ab}}_{3}\right)\right]\times 100$$

Where: Ab_1_ = the absorbance of sample solution.

Ab_2_ = the absorbance of mixture without the enzyme (Blank of sample)

Ab3 = the absorbance of mixture without the sample.

Ab_4_ = the absorbance of mixture only starch without the sample and enzyme.

The results were represented in terms of IC_30_ (µg/mL).

### 2.5 Molecular docking studies

Molecular docking analysis was performed to predict and evaluate the interaction between selected compounds identified from the UHPLC-Q-TOF-MS profile of LSB and clinically relevant diabetic enzymes. This analysis utilized various software tools including PyRx, Autodock Vina, Vina Wizard, Open Babel, and Discovery Studio. The structures of ligands such as spermin, trigonelline, retronecine, quinic acid, lotasutraline, as well as standards acarbose and diprotin-A, were sourced from PubChem (https://pubchem.ncbi.nlm.nih.gov/; accessed on 01 April 2024) in 3-dimensional structure data file (3D-SDF) format. Similarly, receptor molecules including ovine alpha-glucosidase, alpha-amylase, and diprotin-A were obtained from the Protein Data Bank (https://www.rcsb.org/; accessed on 01 April 2024) in PDB format. The preparation of receptor molecules involved tasks such as removing existing ligands and additional chains, eliminating water molecules, and adding polar hydrogens using Discovery Studio (DS) software. Similarly, ligand structures were minimized using the embedded Bebel tool within PyRx. Subsequently, the prepared receptor and ligand structures underwent docking analysis using Autodock Vina. The interactions between receptors and ligands were visualized using Discovery Studio software.

### 2.6 Animals and housing conditions

Seven weeks old healthy littermate male Sprague Dawley rats (180–200 g) were procured from Nomura Siam International (Bangkok, Thailand). The animals were handled humanely throughout the experimental duration. The rats were accommodated in stainless cages in an animal house facility with controlled temperature (22°C ± 2°C), relative humidity (55% ± 10%) and a daily 12 h light/dark cycle. The rats were given unrestricted access to standard rat chow and tap water *ad libitum*. The animals were adapted to the laboratory conditions for seven before the start of the experiment. The ethics committee of the Prince of Songkla University, Hat Yai reviewed and approved the experimental protocol (approval number: 2,564–04–087).

### 2.7 Induction of insulin resistance, diabetes and experimental design

The animals were subsequently allotted into two groups after the acclimatization period and fed with either standard rat chow and tap water or 30% fructose solution together with the standard rat chow for 4 weeks (Olatunji et al., 2023). Thereafter, the rat group fed with the 30% fructose solution were intraperitoneally injected with 40 mg/kg of streptozotocin (dissolved in 0.1 M sodium citrate buffer, pH 4.5) after an overnight fast. In likewise manner, the animals fed with standard rat chow and tap water were also intraperitoneally injected with the same volume of sodium citrate buffer (0.1 M, pH 4.5). Seventy 2 hours post streptozotocin administration, all the animals were evaluated for their fasting blood glucose level with the aid of an Accu-Chek guide glucose monitoring device. Animals were considered diabetic when their fasting blood glucose level was above 250 mg/dL (Olatunji et al., 2017). Thereafter, the rats were randomly assigned into four groups as follows: normal group, diabetic model group, diabetic + LLSB group and diabetic + HLSB group. The rats in the normal and diabetic model groups received distilled water, while the rats in the diabetic + LLSB and diabetic + HLSB groups received 100 and 400 mg/kg of LSB extract, respectively. LSB extract was solubilized in distilled water and administered per os once daily for five consecutive weeks.

Twenty 4 hours after the last treatment, all the rats were subjected to intraperitoneal glucose tolerance test (IPGTT) as previously described (Makinde et al., 2020). In a nutshell, all the animals were given an intraperitoneal injection of glucose (2 g/kg) after an overnight fast and their blood glucose level were determined at time intervals of 0, 30, 60, 90 and 120 min post glucose administration. The area under the curve (AUC) for the IPGTT was calculated using GraphPad Prism. Twenty 4 hours after the IPGTT, the rats were euthanized with sodium thiopental, and blood samples were collected via cardiac puncture for onward centrifugation. The serum obtained was subjected to further biochemical analysis. The pancreas was also collected and separated from adhering tissues. One third of each pancreas tissue was kept in 10% buffer formalin solution for histopathological analysis, while the remaining tissues were preserved at −80°C for further biochemical assays.

### 2.8 Serum biochemical estimation

Using colorimetric method with assays kits, the serum concentrations of glycated haemoglobin, cholesterol, triglyceride, low-density lipoprotein cholesterol, high-density lipoprotein, aspartate aminotransferase, alanine aminotransferase, alkaline phosphatase, blood urea nitrogen and creatinine were determined. Serum fasting insulin level was assayed with ELISA kits as per the manufacturer’s guide (Abcam, Cambridge, UK). Homeostasis Model Assessment of insulin resistance (HOMA-IR), Homeostasis Model Assessment for β cell function (HOMA-β) and Quantitative Insulin Check Index (QUICKI) were calculated using the following equations: HOMA-IR = fasting insulin (μIU/mL) × fasting blood glucose (mg/dL)/405; HOMA-β = [(360 x fasting serum insulin in μIU/mL)/(fasting blood glucose in mg/dL - 63)] and QUICKI = 1/[log(fasting serum insulin in μIU/mL) + log(fasting blood glucose in mg/dL) (Motto et al., 2022).

### 2.9 Estimation of oxido-inflammatory parameters

The levels of inflammatory cytokines including tumor necrosis factor-alpha (TNF-α) and interleukin-1-beta (IL-1β), as well as oxidative stress markers such as superoxide dismutase (SOD) and catalase (CAT), glutathione (GSH) and malonaldehyde (MDA) were estimated in the pancreas. Briefly, the harvested pancreas were washed with ice-cold physiological saline solution. The homogenates of the different pancreas tissues (10% w/v) were made in the phosphate buffer saline. The supernatant obtained after homogenization and centrifugation was used for the estimations of the inflammatory cytokines using commercially available ELISA kits (Abcam, UK) and oxidative stress markers by using commercial biochemical kits (Abbkine, Inc, China) following the guidelines of provided by the manufacturers.

### 2.10 Histopathological and insulin immunohistochemical evaluation

The pancreas tissues from animals in each group preserved with 10% buffered formalin were subjected to dehydration and further treated with paraffin wax for blocking. The microtome sliced sections of the tissues were dyed with hematoxylin and eosin (H&E) and visualized under a microscope equipped with a digital camera for image acquisition.

For insulin immunohistochemical staining of the pancreas, tissue sections were subjected to antigen retrieval, blocking, and the sections were incubated with an anti-insulin antibody overnight at 4°C. Thereafter, the sections were washed and subjected to incubation at 37°C for 1 h with secondary antibodies, washed, treated with diaminobenzidine and further counterstained with hematoxylin. Sections were visualized under a light microscope for image acquisition. The IHC images were analyzed using the ImageJ for insulin positive staining.

### 2.11 Statistical analysis

Data analysis was performed with GraphPad Prism (version 8.0), while comparison among groups was accessed using one-way analysis of variance (ANOVA) and Tukey’s *post hoc* test. *p* values less than 0.05 were considered statistically significant. Data are presented as mean ± standard deviation.

## 3 Results

### 3.1 Total phenolic and flavonoid content

The TPC and TFC of LSB was 54.95 ± 1.69 mg GAE/g and 8.45 ± 0.92 mg of quercetin/g, respectively (Table 1).

**TABLE 1:** Total phenolic and flavonoid content and *in vitro* antioxidant effect of LSB.

Sample	TPC (mg GAE/g d.w.)	TFC (mg QE/g d.w.)	DPPH (IC_50_ µg/mL)	ABTS (IC_50_ µg/mL)	FRAP (QE/g d.w.)
LSB	54.94 ± 1.69	8.45 ± 0.92	578.34 ± 1.77	68.42 ± 2.97	9.85 ± 0.25

LSB: *lepionurus sylvestris* blume extract; QE: quercetin equivalent; GAE: gallic acid equivalent; d.w.: dry weight of the extract.

### 3.2 *In vitro* antioxidant activity

The results of the DPPH, ABTS and FRAP assay are summarized in Table 1. LSB showed DPPH and ABTS inhibitory activity at IC_50_ values of 578.34 ± 1.77 and 68.42 ± 2.97 μg/mL, respectively. Moreover, LSB also showed good ferric reducing ability at a IC_50_ value of 9.85 ± 0.25 mg quercetin per gram of the dried extract (Table 1).

### 3.3 *In vitro* antidiabetic activity

The results of the *in vitro* antidiabetic activity of LSB is shown in Table 2. LSB showed a reasonable α-glucosidase inhibitory activity at an IC_50_ value of 181.65 ± 1.60 μg/mL, higher than the standard acarbose with an IC_50_ value of 634.70 ± 1.56 μg/mL. Whereas, a lower inhibitory activity was observed against α-amylase by showing an IC_30_ value of 4549.68 ± 0.67 μg/mL. Similarly non-significant activity was found against DPP-IV enzyme by showing 18.57% ± 0.94% inhibition at 2,105.26 μg/mL.

**TABLE 2:** *In vitro* antidiabetic effect of LSB.

Sample	Alpha-glucosidase inhibition (IC_50_, µg/mL)	Alpha amylase inhibition (IC_30_, µg/mL)	DPP-IV inhibition (%)
LSB	181.65 ± 1.60	4549.68 ± 0.67	18.57 ± 0.94^a^
Acarbose	634.70 ± 1.56	14.20 ± 3.00	-
Diprotin-A	-	-	99.21 ± 0.29^b^

LSB: *lepionurus sylvestris* blume extract.

^a^ the %inhibition at 2,105.26 μg/mL conc. Of LSB.

^b^ the %inhibition at 61.58 μg/mL conc. of diprotin-A.

### 3.4 UPLC-Q-TOF-MS profiling

The UPLC-Q-TOF-MS metabolite profiling of LSB extract tentatively identified the presence of 77 phytoconstituents, with 64 compounds in positive mode and 13 compounds were detected in negative mode of ionization (Table 3). The major classes of the compounds identified were terpenoids, peptides, coumarins, flavonoids and alkaloids. Several flavonoids and flavonoid glycosides were putatively identified in LSB. For instance, compound #65 was putatively identified as the flavanol kaempferol (Rt = 8.622 min, m/z 287.0556), while compound #47 was established as scutellarein. Additionally, several flavonoid derivatives putatively identified in LSB includes compound #60 kaempferol-3-hexoside (Rt = 8.205 min), compound #61 genistein -7-*O*-Glc-Xyl, acetate (Rt = 8.24 min), compound #64 cyanidin-3-*O*-α-arabinoside (Rt = 8.622 min), compound #70 2′-hydroxy-a-naphthoflavone (Rt = 11.453 min), compound #71 6,4′-dimethoxyisoflavone-7-glucoside (Rt = 14.301) and compound #73 hispiduloside (Rt = 16.856). The UPLC-Q-TOF-MS data putatively identified the presence of the following terpenoids; nardosinone (Rt = 0.801 min), myrcene (Rt = 1.301 min), germacrone (Rt = 2.156 min), soyasapogenol A (Rt = 6.139 min), dehydroabietamide (Rt = 6.495 min), jolkinolide B (Rt = 7.107 min) and ziyuglycoside II (Rt = 9.164 min). Furthermore, a number of alkaloids were also prominently identified in LSB, including trigonelline (Rt = 0.974 min), oxysophocarpine (Rt = 1.267 min), retronecine (Rt = 1.284 min), ecgonine (Rt = 1.573 min) and homatropine (Rt = 6.669 min).

**TABLE 3:** Tentative identities of compounds in LSB profiled by LC-ESI-QTOF-MS analysis.

No.	Rt (min)	Mass detected	Tentative identification	Adduct type	Formula	Ontology
1	0.801	251.16022	Nardosinone	[M + H]^+^	C_15_H_22_O_3_	Sesquiterpenoid
2	0.883	218.93314	4-Iodphenol	[M-H]^-^	C_6_H_5I_O	P-iodophenol
3	0.89	180.97858	4-*O*-Methylphloracetophenone	[M-H]^-^	C_9_H_10_O_4_	Alkyl-phenylketone
4	0.904	205.06815	Sorbitol	[M + H]^+^	C_6_H_14_O_6_	Sugar alcohol
5	0.974	138.05508	Trigonelline	[M + H]^+^	C_7_H_7_NO_2_	Alkaloid
6	0.991	262.1311	Lotaustralin	[M + H]^+^	C_11_H_19_NO_6_	Cyanogenic glycoside
7	0.991	193.07025	Quinic acid	[M + H]^+^	C_7_H_12_O_6_	Cyclitol
8	1.025	207.06519	Scoparone	[M + H]^+^	C_11_H_10_O_4_	Coumarin
9	1.242	112.05074	Cytosine	[M + H]^+^	C_4_H_5_N_3_O	Nucleoside
10	1.267	263.17145	Oxysophocarpine	[M + H]^+^	C_15_H_22_N_2_O_2_	Alkaloid
11	1.284	146.09273	4-Guanidinobutyric acid	[M + H]^+^	C_5_H_11_N_3_O_2_	Alkaloid
12	1.284	130.08736	Pipecolic acid	[M + H]^+^	C_6_H_11_NO_2_	Amino acid
13	1.284	156.1021	Retronecine	[M + H]^+^	C_8_H_13_NO_2_	Alkaloid
14	1.301	159.12422	Myrcene	[M + H]^+^	C_10_H_16_	Monoterpenoid
15	1.326	139.00276	Fumaric acid	[M + H]^+^	C_4_H_4_O_4_	Carboxylic acid
16	1.573	186.11214	Ecgonine	[M + H]^+^	C_9_H_15_NO_3_	Tropane alkaloid
17	1.696	136.07472	2-Phenylacetamide	[M + H]^+^	C_8_H_9_NO	Phenylacetamide
18	1.77	268.10379	Adenosine	[M + H]^+^	C_10_H_13_N_5_O_4_	Nucleoside
**19**	2.156	241.15482	Germacrone	[M + H]^+^	C_15_H_22_O	Sesquiterpenoid
20	2.804	643.41632	Ginsenoside Rh4	[M + H]^+^	C_36_H_60_O_8_	Triterpenoid
21	3.239	149.05983	p-Coumaraldehyde	[M + H]^+^	C_9_H_8_O_2_	Cinnamaldehyde
22	5.125	208.13376	Phenylalanine betaine	[M + H]^+^	C_12_H_17_NO_2_	Phenylalanine
23	5.761	627.42383	Ginsenoside Rh3	[M + H]^+^	C_36_H_60_O_7_	Triterpenoid
24	6.139	475.37262	Soyasapogenol A	[M + H]^+^	C_30_H_50_O_4_	Triterpenoid
25	6.267	129.13913	Spermine	[M + H]^+^	C_10_H_26_N_4_	Dialkylamine
26	6.303	145.02872	Benzoic acid	[M + H]^+^	C_7_H_6_O_2_	Benzoic acid
27	6.319	492.90015	3,5-Dichlororsellinic acid	[M-H]^-^	C_8_H_6_C_l2_O_4_	Dichlorobenzoic acid
28	6.387	159.09181	1,5-Naphthalenediamine	[M + H]^+^	C_10_H_10_N_2_	Naphthalene
29	6.495	439.21994	N-(3-(dimethylamino)propyl)-2-((3,4,8,8-tetramethyl-2-oxo-2,8,9,10-tetrahydropyrano [2,3-f]chromen-5-yl)oxy)acetamide	[M + H]+	C_23_H_32_N_2_O_5_	Angular pyranocoumarin
30	6.495	373.19824	3,3′,4,4′-tetramethoxy-7,7′-epoxylignan	[M+2H]^2+^	C_22_H_28_O_5_	7,7′-epoxylignans
31	6.495	322.21323	Dehydroabietamide	[M + H]^+^	C_20_H_29_NO	Diterpenoid
32	6.53	167.07082	Paeonol	[M + H]^+^	C_9_H_10_O_3_	Phenol
33	6.669	265.15485	Feruloyl putrescine	[M + H]^+^	C_14_H_20_N_2_O_3_	Hydroxycinnamic acid
34	6.669	276.15805	Homatropine	[M+2H]^2+^	C_16_H_21_NO_3_	Tropane alkaloid
35	6.716	261.14423	Gamma-Glutamylleucine	[M + H]^+^	C_11_H_20_N_2_O_5_	Dipeptide
36	6.75	161.10771	2-(1H-indol-3-yl)ethanamine	[M + H]^+^	C_10_H_12_N_2_	Tryptamine
37	6.767	229.15508	Leucylproline	[M + H]^+^	C_11_H_20_N_2_O_3_	Dipeptide
38	6.835	231.17047	Leucylvaline	[M + H]^+^	C_11_H_22_N_2_O_3_	Dipeptide
39	6.868	322.18182	Gabexate	[M+2H]^2+^	C_16_H_23_N_3_O_4_	Benzoic acid ester
40	6.886	298.15277	Eserine	[M+2H]^2+^	C_15_H_21_N_3_O_2_	Alkaloid
41	6.901	147.04424	Coumarin	[M + H]^+^	C_9_H6O_2_	Coumarin
42	6.922	290.9953	4-Chloronorlichexanthone	[M-H]^-^	C_14_H_9_ClO_5_	Xanthone
43	7.04	290.1554	Brevicarine	[M+2H]^2+^	C_17_H_21_N_3_	Alkaloid
44	7.107	331.18677	Jolkinolide B	[M+2H]^2+^	C_20_H_26_O_4_	Diterpenoid
45	7.107	400.25635	Cryptomaldamide	[M + H]^+^	C_18_H_33_N_5_O_5_	Peptide
46	7.123	177.05493	Ferulic acid	[M + H]^+^	C_10_H_10_O_4_	Hydroxycinnamic acid
47	7.369	287.13907	Scutellarein	[M + H]^+^	C_15_H_10_O_6_	Flavone
48	7.417	303.16986	Piperine	[M + H]^+^	C_17_H_19_NO_3_	Alkaloid
49	7.569	245.18567	Leucylleucine	[M + H]^+^	C_12_H_24_N_2_O_3_	Peptide
50	7.584	286.17093	Dendrobine	[M+2H]^2+^	C_16_H_23_NO_2_	Alkaloid
51	7.597	307.17709	Feruloyl agmatine	[M + H]^+^	C_15_H_22_N4O_3_	Cinnamamide
52	7.629	337.21335	Pregn-4-ene-3,20-dione	[M + H]^+^	C_21_H_30_O_2_	Steroid
53	7.689	337.18716	Catharanthine	[M + H]^+^	C_21_H_24_N_2_O_2_	Alkaloid
54	7.767	743.40662	Stylostatin 1	[M + H]^+^	C_36_H_54_N_8_O_9_	Oligopeptide
55	7.774	243.134	PyroglutamylIsoleucine	[M + H]^+^	C_11_H_18_N_2_O_4_	Dipeptide
56	7.835	380.9783	Eriodermin	[M-H]^-^	C_17_H_12_Cl_2_O_6_	Depsidone
57	7.985	306.16852	Anisodamine	[M+2H]^2+^	C_17_H_23_NO_4_	Tropane alkaloid
58	8.052	279.17035	Leucylphenylalanine	[M + H]^+^	C_15_H_22_N_2_O_3_	Dipeptide
59	8.069	299.1611	6-Shogaol	[M+2H]^2+^	C_17_H_24_O_3_	Shogaol
60	8.205	449.10864	Kaempferol-3-hexoside	[M + H]^+^	C_21_H_20_O_11_	Flavonoid glycoside
61	8.24	901.24811	Genistein -7-O-Glc-Xyl, acetate	[M+2H]^2+^	C_42_H_44_O_22_	Flavonoid glycoside
62	8.268	434.99738	Erigeroside	[M-H]^-^	C_11_H_14_O_8_	O-glycosyl compound
63	8.494	433.12964	Asperulosidic acid	[M + H]^+^	C_18_H_24_O_12_	Iridoid glycoside
64	8.622	419.09814	Cyanidin-3-O-alpha-arabinoside	[M + H]^+^	C_20_H_19_O_10_	Flavonoid glycoside
65	8.622	287.0556	Kaempferol	[M + H]^+^	C15H10O6	Flavonol
66	8.878	603.20691	Episyringaresinol 4′-O-beta-D-glncopyranoside	[M + H]^+^	C_28_H_36_O_13_	Lignan glycoside
67	8.986	495.18887	Harpagoside	[M + H]^+^	C_24_H_30_O_11_	Iridoid glycoside
68	9.164	627.3761	Ziyuglycoside II	[M + H]^+^	C_35_H_56_O_8_	Triterpenoid
69	11.227	329.06165	Nornotatic acid	[M-H]^-^	C_17_H_14_O_7_	Depsidone
70	11.453	287.07263	2′-Hydroxy-a-naphthoflavone	[M-H]^-^	C_19_H_12_O_3_	Flavone
71	14.301	296.99814	6,4′-Dimethoxyisoflavone-7-glucoside	[M-H]^-^	C_23_H_24_O_10_	flavonoid glycoside
72	15.111	311.00635	Hedysarimcoumestan A	[M-H]^-^	C_17_H_12_O_6_	Coumestan
73	16.856	485.11594	Hispiduloside	[M + H]^+^	C_22_H_22_O_11_	Flavonoid glycoside
74	17.739	439.32162	Tigogenin	[M + H]^+^	C_27_H_44_O_3_	Triterpenoid
75	18.655	451.2825	Celastrol	[M + H]^+^	C_29_H_38_O_4_	Triterpenoid
76	18.779	433.22348	Schizandrol A	[M + H]^+^	C_24_H_32_O_7_	Tannin
77	25.081	179.03578	6,7-Dihydroxycoumarin	[M + H]^+^	C_9_H_6_O_4_	Coumarin

### 3.5 Molecular docking

**FIGURE 1:**
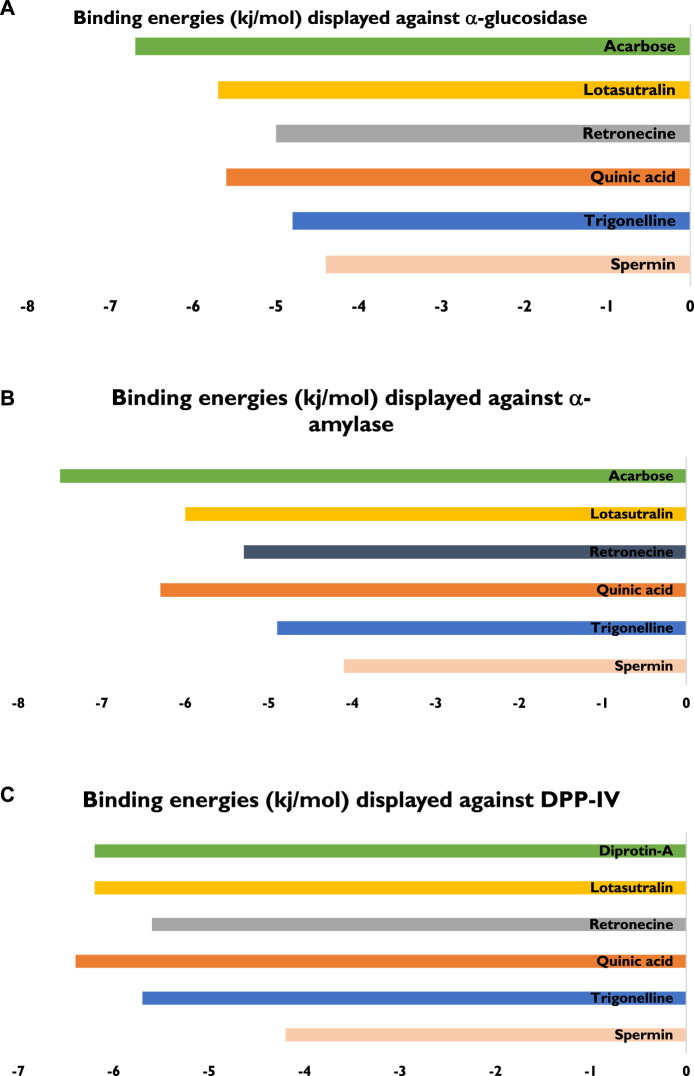
Details of bindings affinities shown by various compounds from UPLC-Q-TOF-MS profile of LSB against **(A)** α-glucosidase **(B)** α-amylase **(C)** DPP-IV enzymes.

**FIGURE 2:**
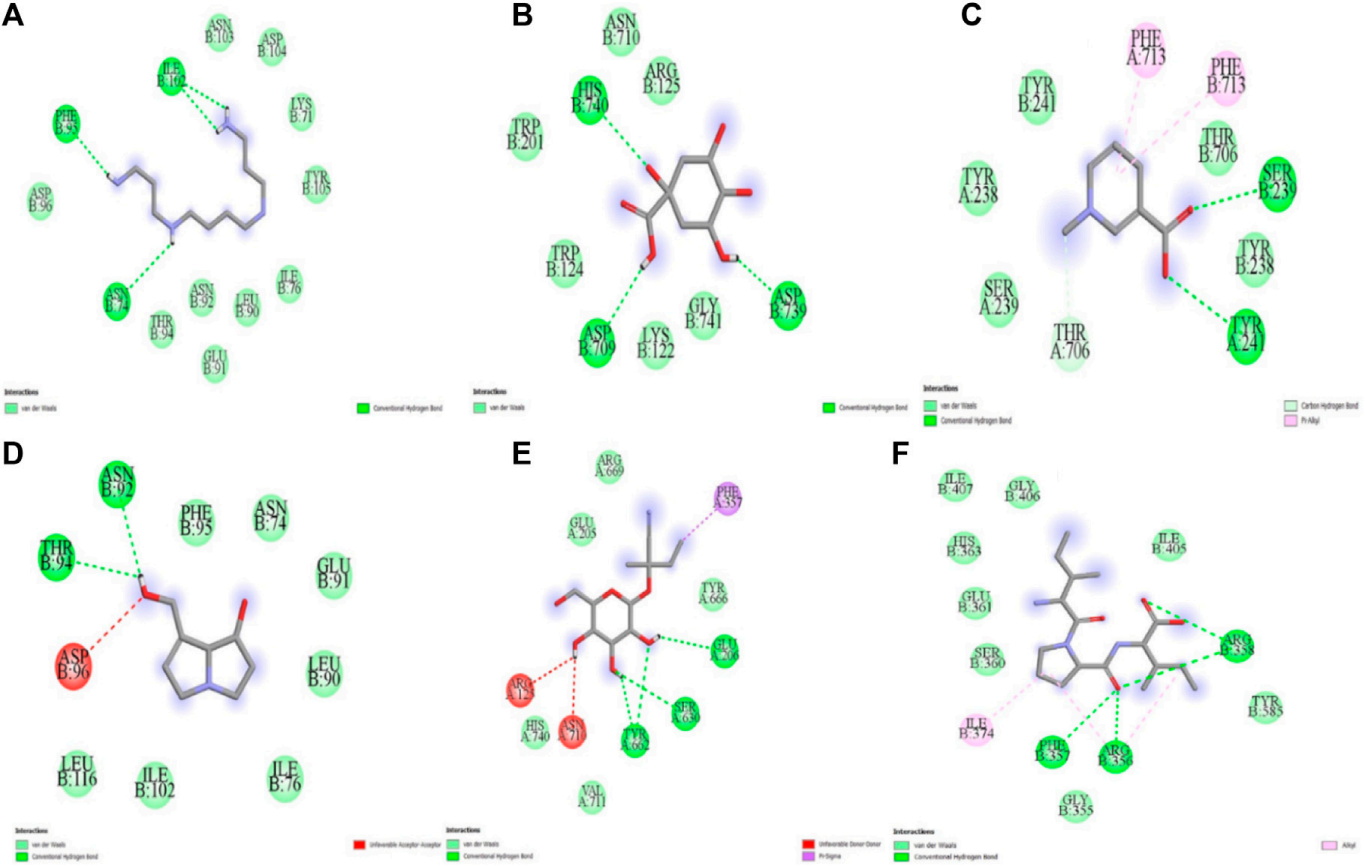
Molecular docking analysis of **(A)** spermin **(B)** quinic acid **(C)** trigonelline **(D)** retronecine **(E)** lotaustralin **(F)** diprotine A against DPP-IV.

**FIGURE 3:**
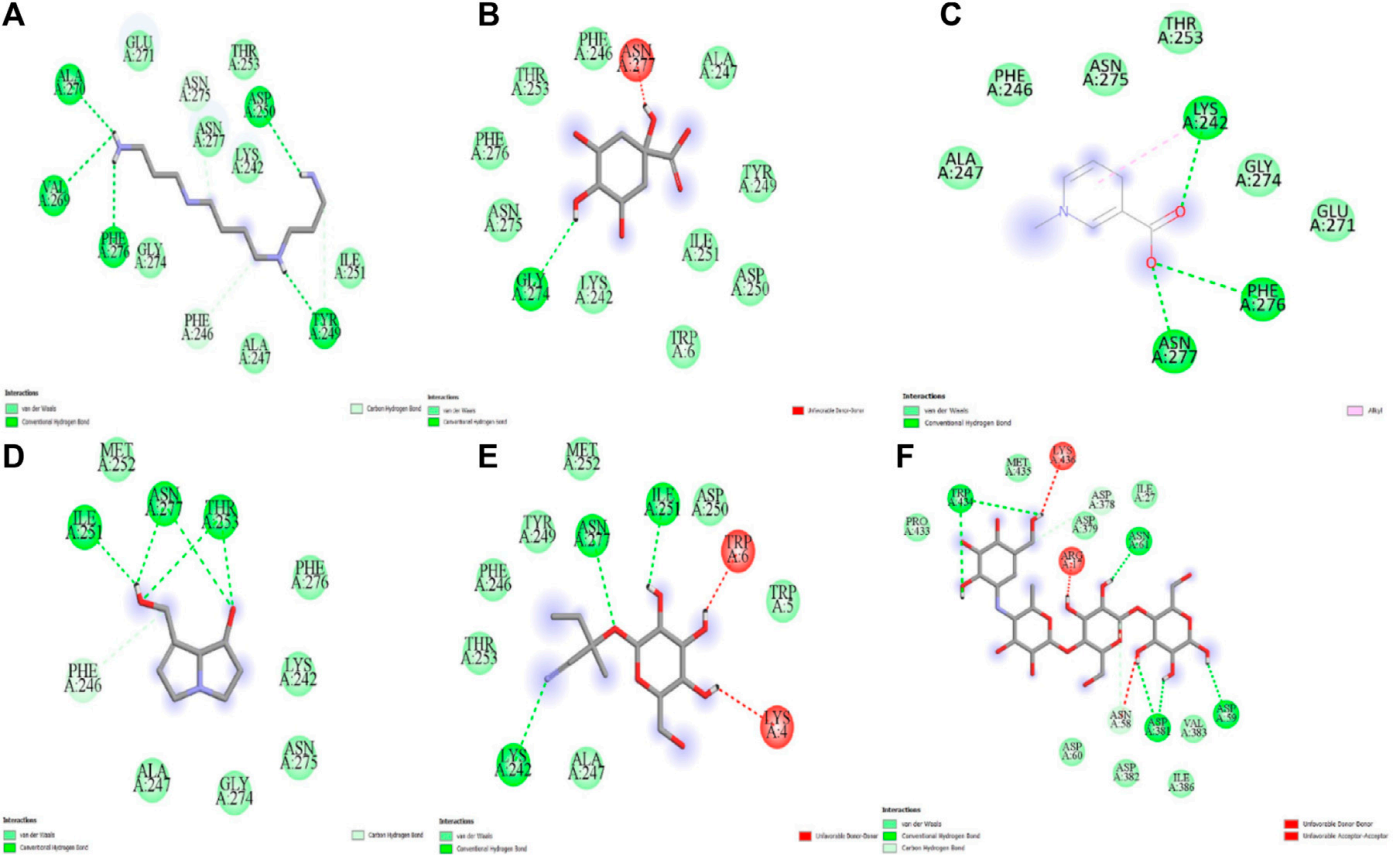
Molecular docking analysis of **(A)** spermin **(B)** quinic acid **(C)** trigonelline **(D)** retronecine **(E)** lotaustralin **(F)** acarbose against α-glucosidase.

**FIGURE 4:**
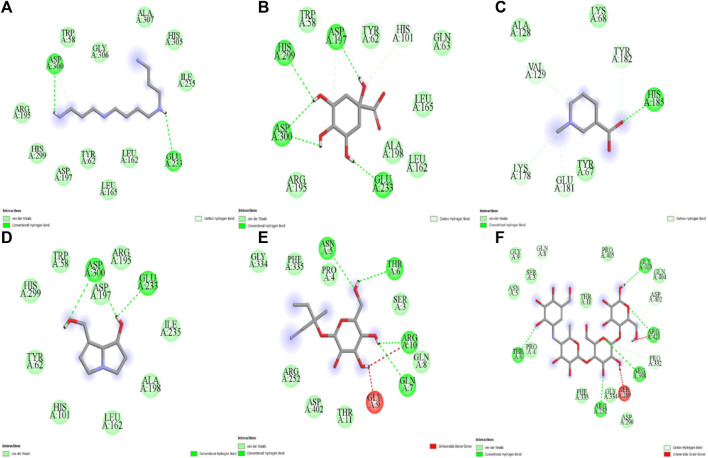
Molecular docking analysis of **(A)** spermin **(B)** quinic acid **(C)** trigonelline **(D)** retronecine **(E)** lotaustralin **(F)** acarbose against α-amylase.

The five compounds spermin, trigonelline, retronecine, quinic acid, lotasutraline were subjected to *in silico* molecular docking study against the clinically significant enzymes α-glucosidase, α-amylase and DPP-IV. The compounds selection was based on the best docking score of the compounds against the enzymes. The findings showed that the compounds displayed varying degree of binding energies (kJ/mol) against the studied enzymes (Figures 1A–C). Lotaustralin showed highest binding affinity and lowest binding energy of −5.7 kJ/mol against α-glucosidase among the compounds but lower than the standard acarbose which displayed a binding energy of −6.7 kJ/mol. Lotaustralin interacts with α-glucosidase through the formation of hydrogen bonds with various amino acid residues of the enzymes (Figure 2). In the case of α-amylase quinic acid was found with highest binding affinity and lowest binding energy of −6.3 kJ/mol but lower than standard acarbose (−7.5 kJ/mol). Figure 3 shows quinic acid interaction with α-amylase by the formation of hydrogen bonding which is why it showed highest binding affinity compared to other compounds. Similarly, in quinic acid showed highest binding affinity for DDP-IV by displaying binding energy of 6.4 kJ/mol slightly higher than standard diprotin-A (6.2 kJ/mol). Figure 4 shows the interaction of quinic acid and DPP-IV through hydrogen bonding which resulted in the lower binding energy and higher binding affinity.

### 3.6 LSB attenuated altered metabolic parameters

**FIGURE 5:**
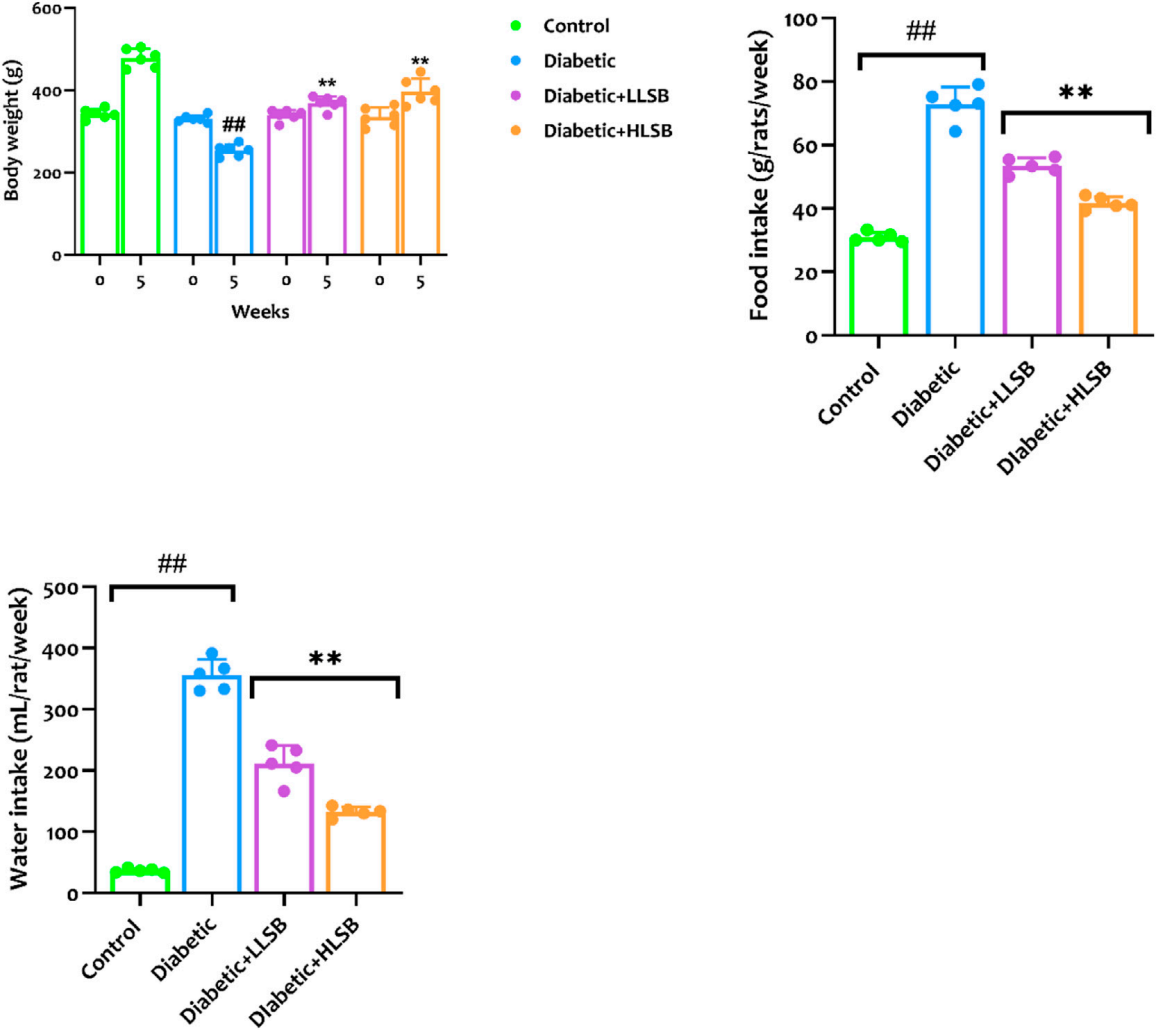
The effect of LSB on body weight gain, food and water consumption in fructose/STZ induced diabetic rats. Values are expressed as mean ± SD of six replicates and were analyzed by one-way ANOVA followed by Tukey’s *post hoc* test. ##*p* < 0.05 vs. healthy control group, **p < 0.05 vs. diabetic model group.

The evaluation of the growth and metabolic parameters are displayed in Figure 5. It was obvious that the food and water consumption as well as the body weight among all the treatment groups were significantly varied. The food and water intake in the diabetic model group were remarkably increased when juxtaposed to the healthy control rats. Likewise, the body weight of the animals in the diabetic model group was observably reduced in comparison with the healthy group (Figure 5). However, treatment with LSB notably increased the final body weight of the diabetic treated rats, while the food intake and water consumption were significantly lowered compared with the diabetic model group (Figure 5).

### 3.7 LSB attenuated glucose homeostasis, increased insulin secretion and beta cell function

**FIGURE 6:**
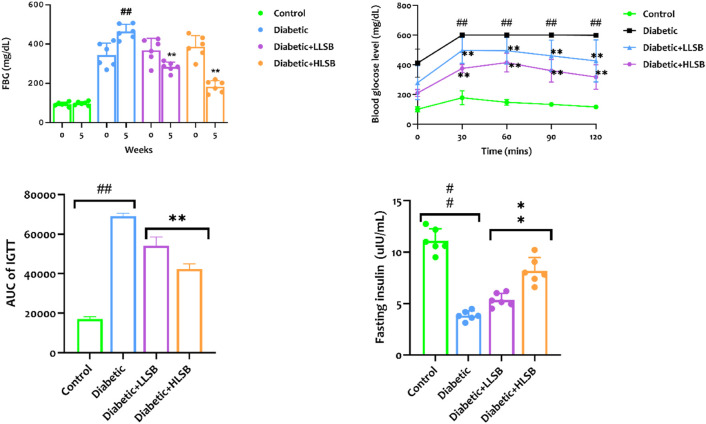
The effect of LSB on fasting blood glucose level, IPGTT, IPGTT AUC and fasting insulin level in fructose/STZ induced diabetic rats. Values are expressed as mean ± SD of six replicates and were analyzed by one-way ANOVA followed by Tukey’s *post hoc* test. ##*p* < 0.05 vs. healthy control group, **p < 0.05 vs. diabetic model group.

The fasting blood glucose levels of all the rats in the diabetic model, LLSB and HLSB groups (343.8–386.3 mg/dL) were significantly higher than the healthy control rats (94.0 mg/dL) before the commencement of the intervention (week 0). On the contrary, the LSB-treated diabetic groups showed remarkable decrease in the fasting blood glucose levels compared to the diabetic model group. The blood glucose level of the LLSB and HLSB groups after 5 weeks of treatment were 283.6 and 191.7 mg/dL, respectively which was notably lower when juxtaposed with the diabetic model rats (410.8 mg/dL, Figure 6).

**FIGURE 7:**
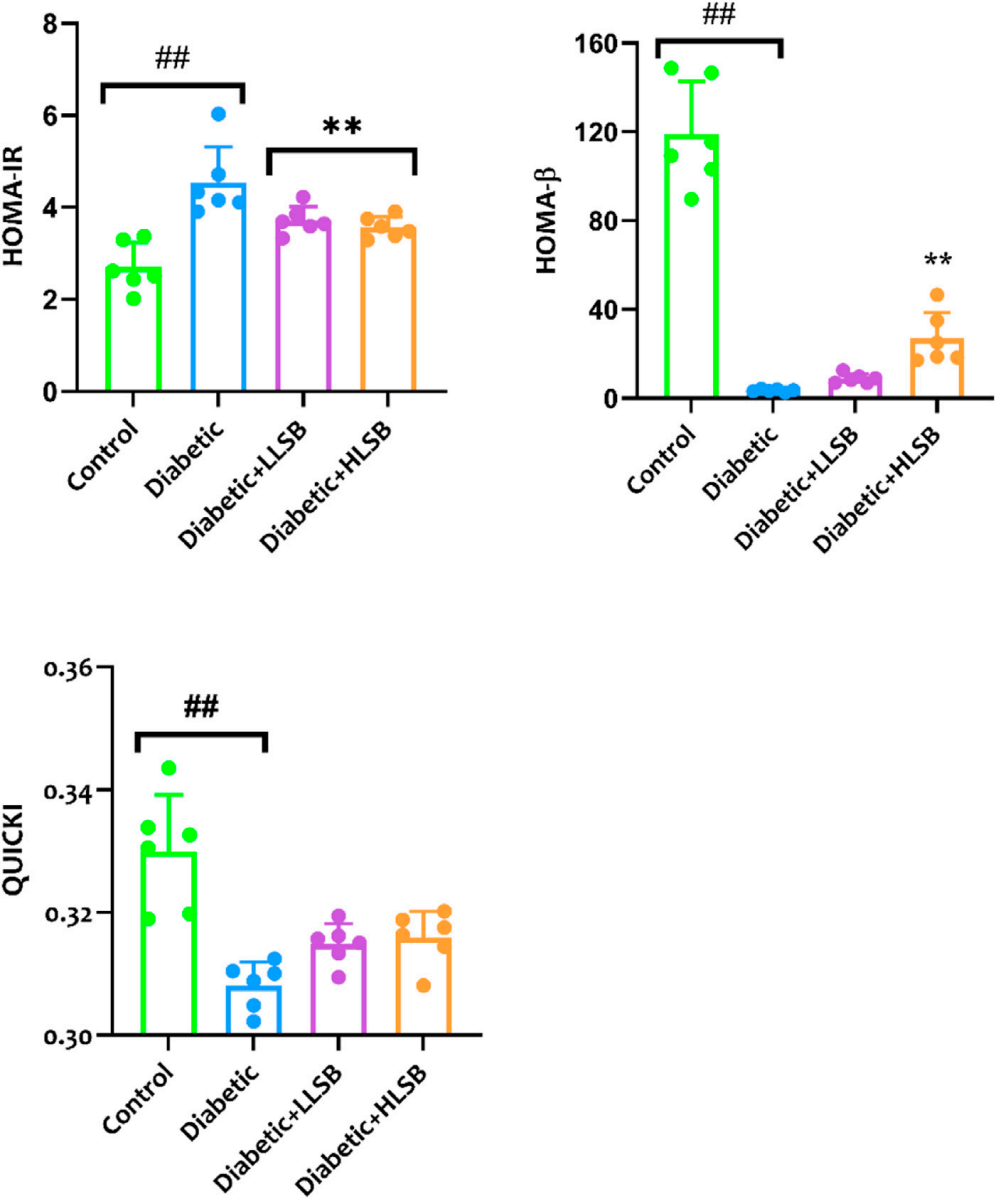
The effect of LSB on HOMA-IR, HOMA-β and QUICKI in fructose/STZ induced diabetic rats. Values are expressed as mean ± SD of six replicates and were analyzed by one-way ANOVA followed by Tukey’s *post hoc* test. ##*p* < 0.05 vs. healthy control group, **p < 0.05 vs. diabetic model group.

In addition, we evaluated the effect of LSB on glucose clearance in the IPGGT by evaluating the blood glucose level at different time points. It was observed that the blood glucose level of all the groups showed an initial increase, reaching the highest peak at 60 min. The blood glucose level of the diabetic model group was consistently and significantly higher than the healthy control and LSB treated groups (Figure 6). Furthermore, the AUC for blood glucose levels in the IPGTT was also notably increased in the diabetic model animals in comparison to the healthy control animals. Notably, LLSB and HLSB treated groups revealed significantly lowered blood glucose levels than the diabetic model group, suggesting improved glucose clearance in the treated animals. In addition, there was obvious reduction in the fasting insulin level of the diabetic model group compared to the healthy control group. Whereas, both treatment doses of LSB significantly elevated the serum insulin concentration in comparison with the diabetic model group. Furthermore, the HOMA-IR values indicated that insulin resistance was notably increased in the diabetic model group when compared to the healthy control animals, while LSB could significantly decreased insulin resistance as observed in the reduced levels of HOMA-IR in the treated rats. There were no significant difference between the diabetic control and LSB treated groups with respect to QUICKI insulin sensitivity. However, there was a significant in HOMA-β values of the diabetic rats when compared with normal controls and HLSB treated groups (Figure 7).

### 3.8 H&E and IHC staining of the pancreas

**FIGURE 8:**
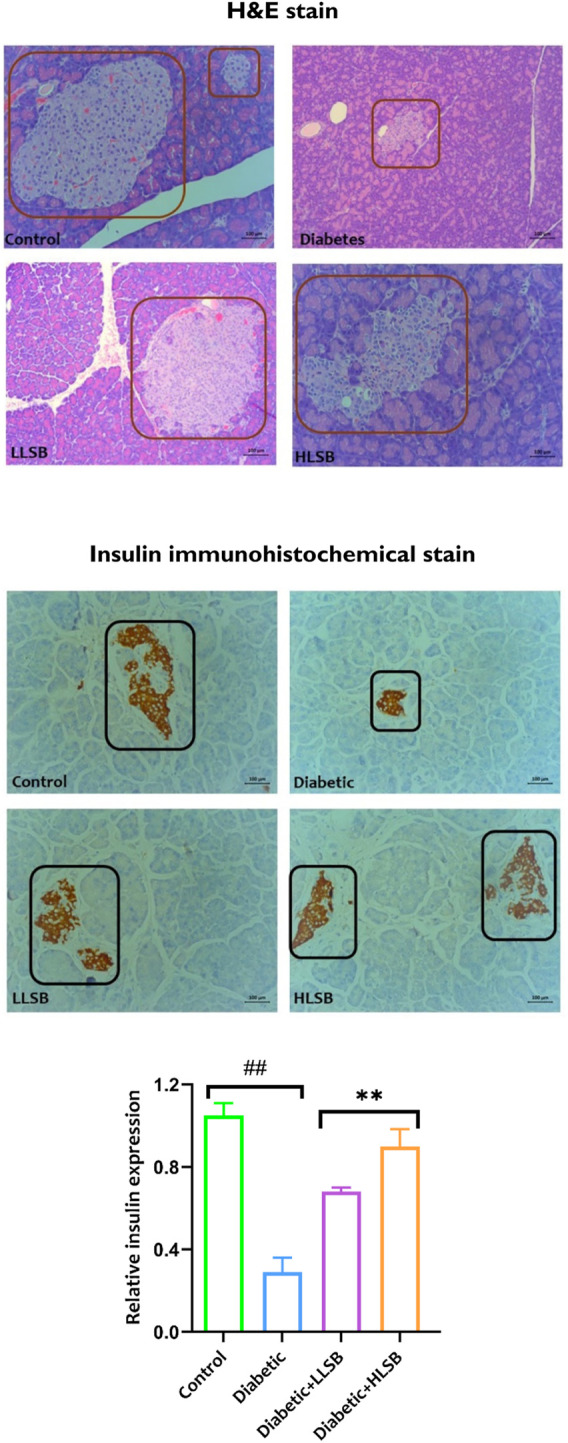
The effect of LSB on pancreas histopathology and insulin immunohistochemical staining in fructose/STZ induced diabetic rats. Magnification ×200.

Histological examination of the pancreatic tissues architecture using H&E staining demonstrated that the diabetic model group showed decrease in the number and size of pancreatic beta cells compared with the healthy control group (Figure 8). In addition, pancreatic hyperplasia was observed in the tissues of the diabetic model group. Both LLSB and HLSB treatments alleviated pancreatic architecture damage compared to the diabetic model group, as portrayed by increase in the size and number of beta cells of Langerhans (Figure 8). Furthermore, in the IHC staining for insulin, it was observed that the diabetic model group showed reduced brown stain intensity, suggesting decreased insulin immunoreactivity in comparison to the healthy control and LSB treated groups (Figure 8).

### 3.9 LSB attenuated altered biochemical parameters

**FIGURE 9:**
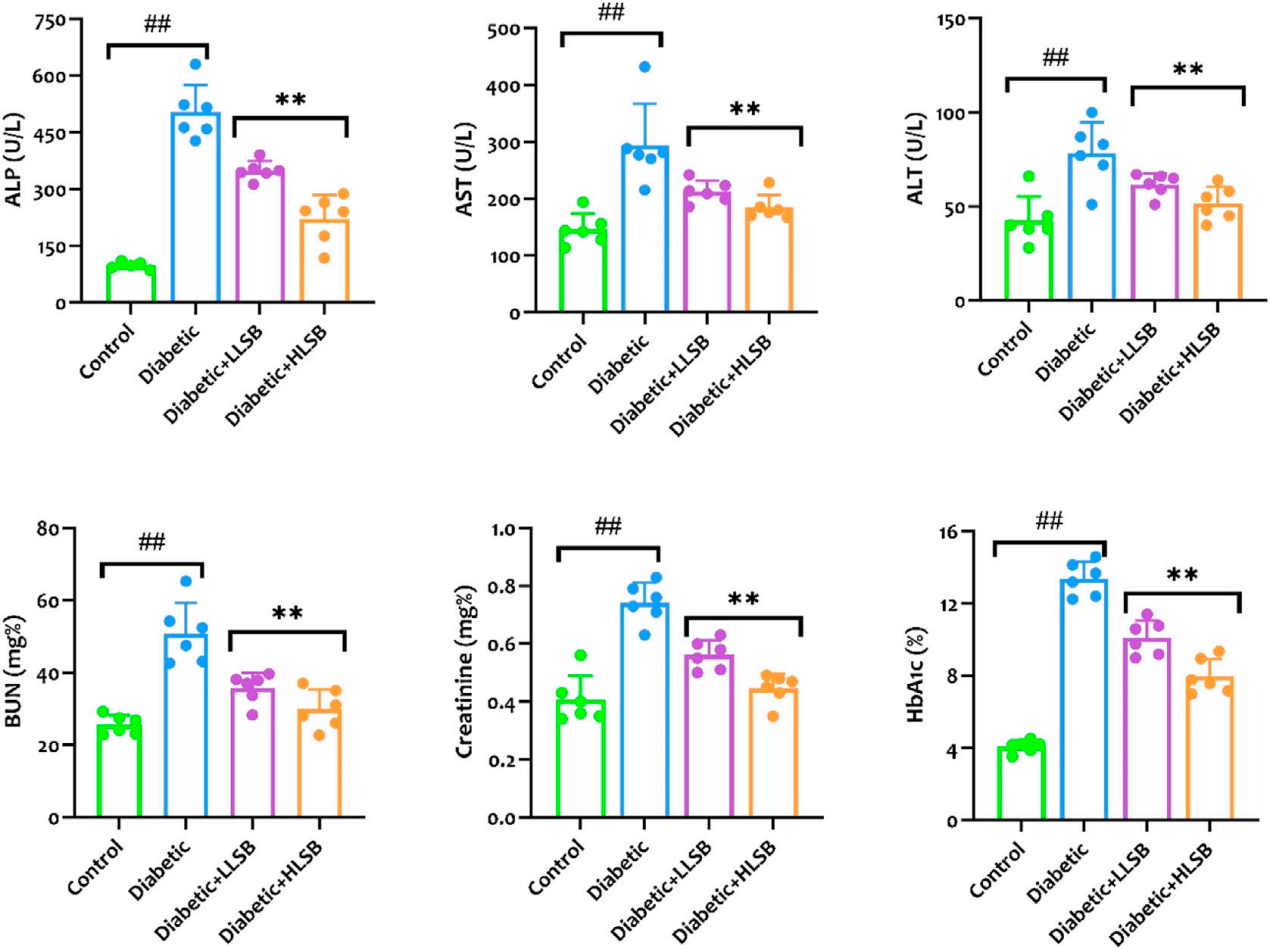
The effect of LSB on serum ALP, AST, ALT, BUN, creatinine and HbA1c in fructose/STZ induced diabetic rats. Values are expressed as mean ± SD and were analyzed by one-way ANOVA followed by Tukey’s *post hoc* test. ##*p* < 0.05 vs. healthy control group, **p < 0.05 vs. diabetic model group.

**FIGURE 10:**
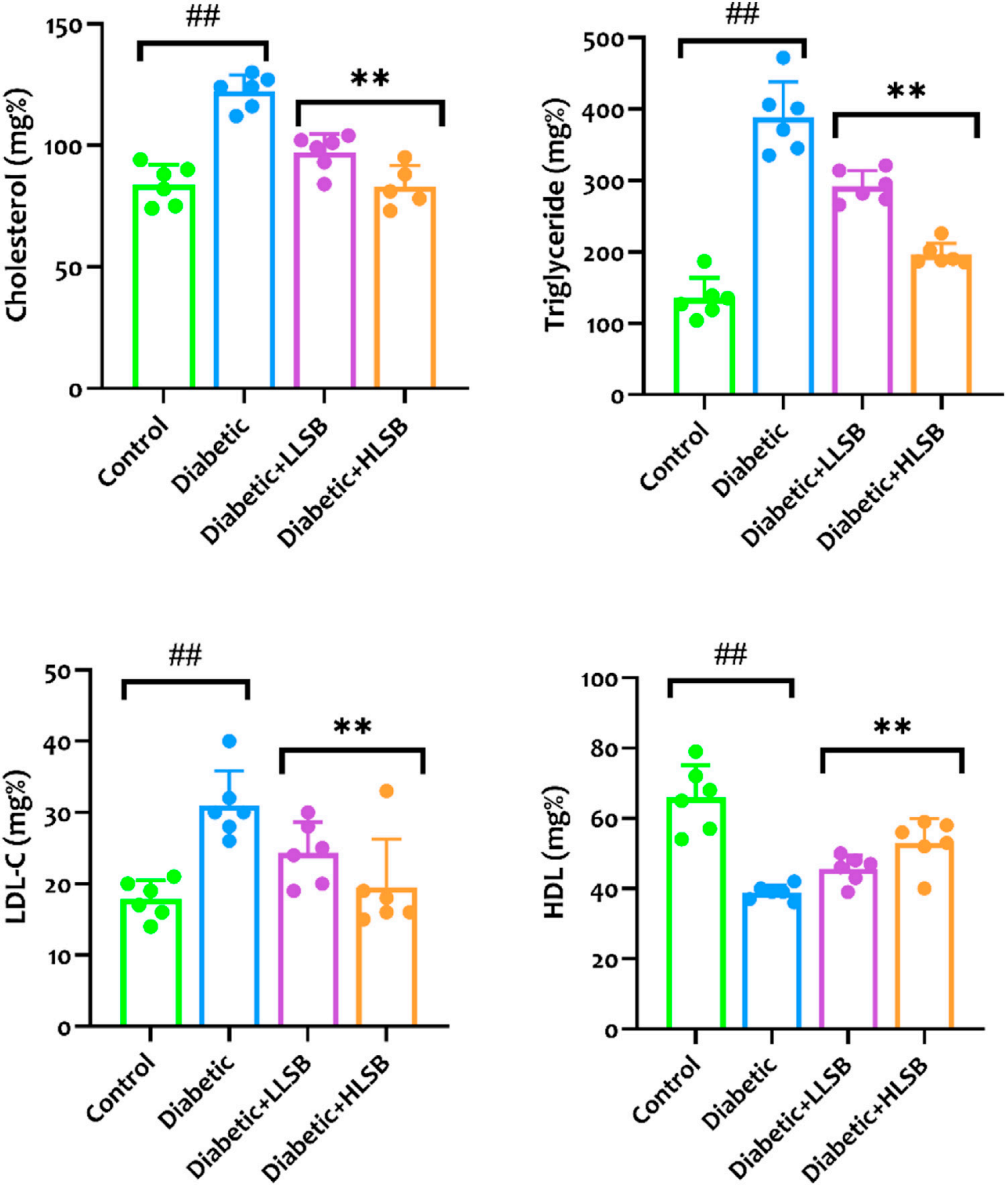
The effect of LSB on serum cholesterol, triglyceride, low density lipoprotein cholesterol and high density lipoprotein in fructose/STZ induced diabetic rats. Values are expressed as mean ± SD and were analyzed by one-way ANOVA followed by Tukey’s *post hoc* test. ##*p* < 0.05 vs. healthy control group, **p < 0.05 vs. diabetic model group.

Measurement of the serological hepatorenal markers demonstrated marked elevation in the levels of BUN, creatinine, ALP, AST and ALT of the diabetic model rats compared to the healthy control, while treatment with LSB significantly abated the levels of the hepatorenal function markers (Figure 9). Furthermore, the serum HbA1c levels were substantially elevated in the diabetic model rats as compared with the healthy control group, while the groups treated with LSB showed significant dose-related decrease in HbA1c levels in comparison to the diabetic rats. In addition, treatment with LSB led to significant decreases in the serum TC, TG and LDL-c levels in comparison with the diabetic model group. Serum HDL accumulation in the diabetic model rats was observed to be markedly reduced in comparison with healthy control, whilst on the contrary, treatment with LLSB and HLSB restored HDL levels when compared with the diabetic model group (Figure 10).

### 3.10 LSB attenuated pancreatic oxidative stress markers

**FIGURE 11:**
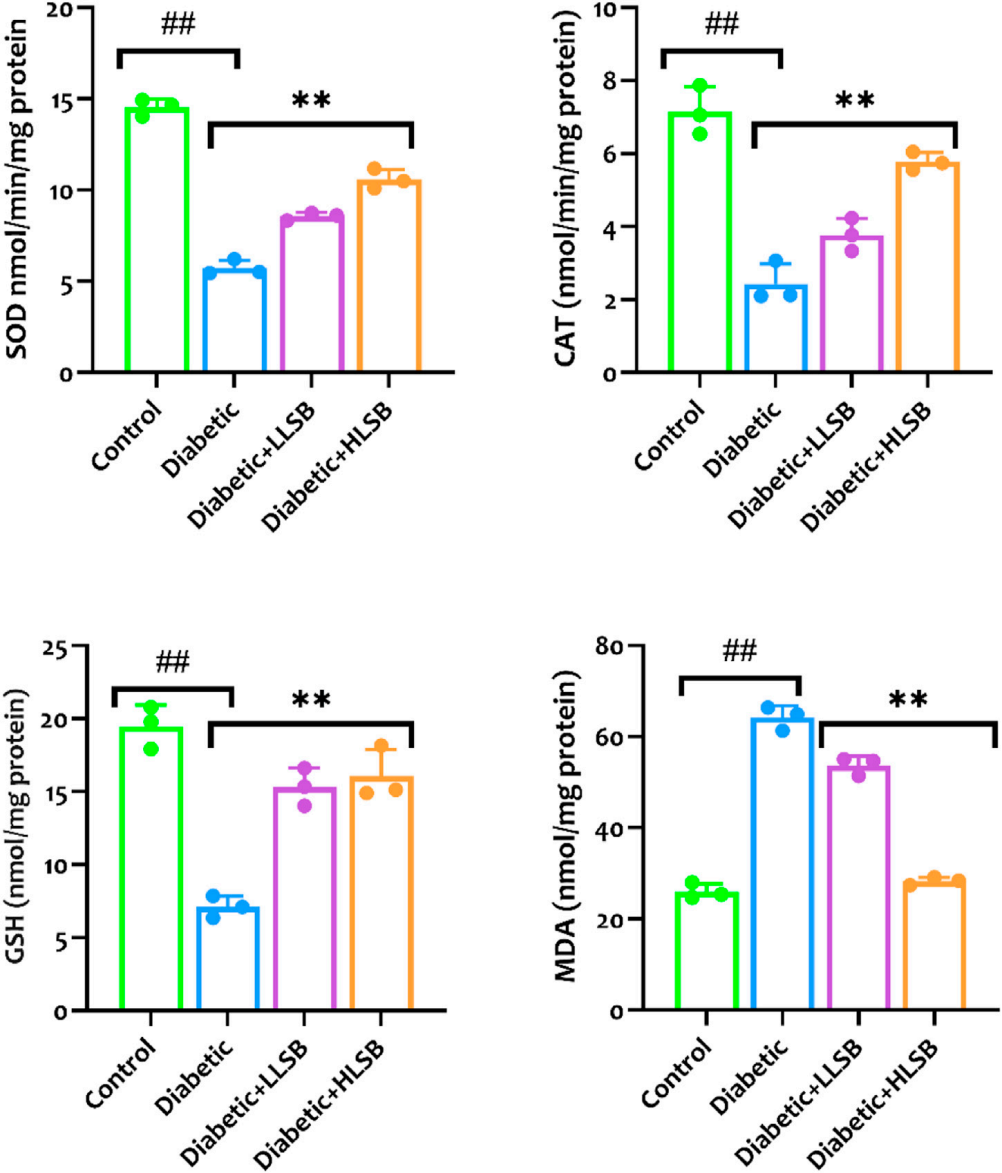
The effect of LSB on pancreas SOD, CAT, GSH and MDA in fructose/STZ induced diabetic rats. Values are expressed as mean ± SD and were analyzed by one-way ANOVA followed by Tukey’s *post hoc* test. ##*p* < 0.05 vs. healthy control group, **p < 0.05 vs. diabetic model group.

As portrayed in Figure 11, the diabetic rats treated with LSB showed significantly elevated levels of pancreatic GSH, SOD and CAT when compared to the diabetic model rats with profound decreases in the GSH, SOD and CAT levels. The diabetic model group showed a significant decline in GSH, SOD and CAT levels by 63.6, 59.9% and 66.1%, respectively when compared to the healthy control group (Figure 11). In contrast, a significant increase in the pancreatic MDA level by 58.5% was observed in the diabetic model rats as compared with the healthy control rats, while treatment with LLSB and HLSB recorded significant reduction in pancreatic MDA level by 16.4% and 56.9%, respectively when juxtaposed with the model rats (Figure 11).

### 3.11 LSB attenuated pancreatic pro-inflammatory cytokines

**FIGURE 12:**
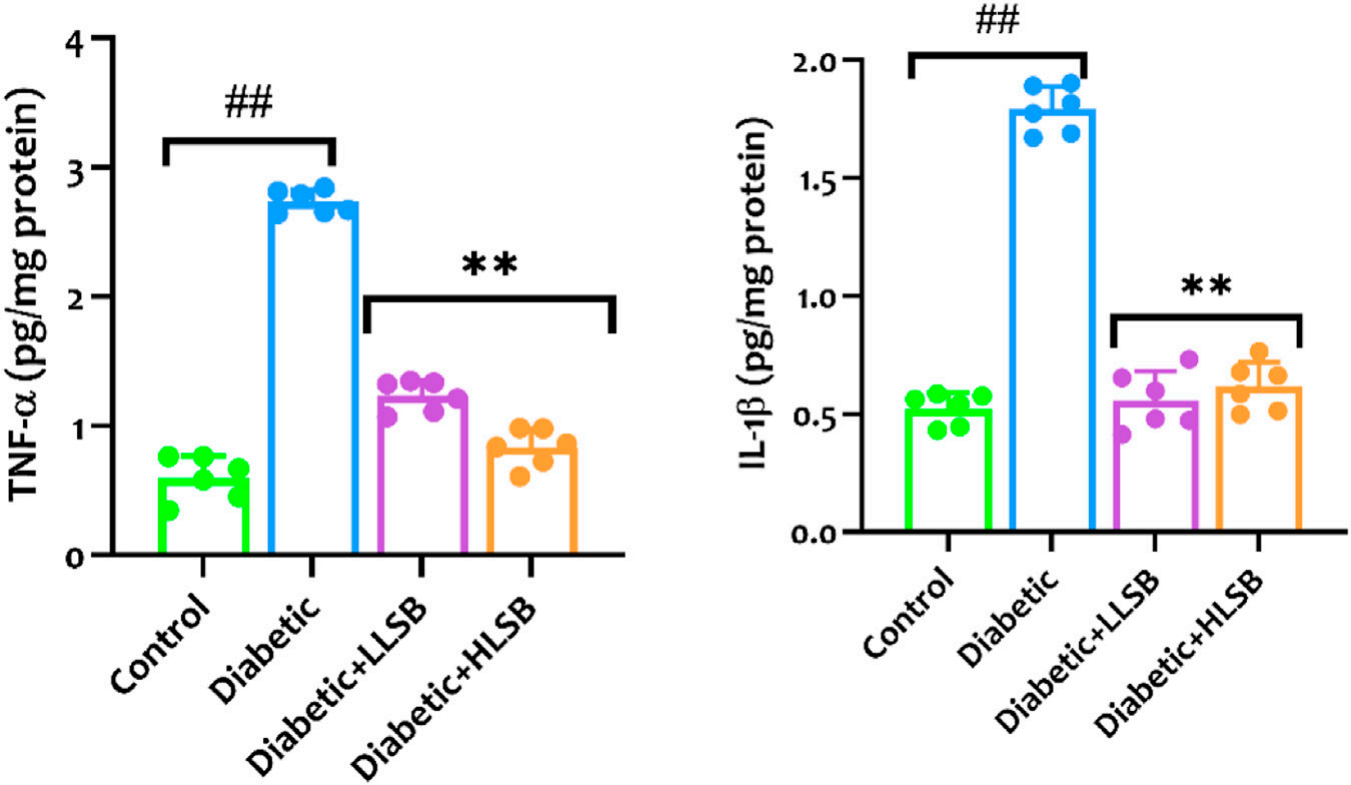
The effect of LSB on pancreas TNF-α and IL-1β in fructose/STZ induced diabetic rats. Values are expressed as mean ± SD and were analyzed by one-way ANOVA followed by Tukey’s *post hoc* test. ##*p* < 0.05 vs. healthy control group, **p < 0.05 vs. diabetic model group.

As shown in Figure 12, the levels of proinflammatory cytokines (IL-1β and TNF-α) in the pancreatic tissues of rats in the diabetic model group were significantly higher than that of the healthy control group. Whereas, compared with the model group, pancreatic IL-1β was reduced dose dependently by 68.8% and 65.6%, while TNF-α level was decreased by 55.0% and 69.6%, respectively in the LSB treated groups (Figure 12).

## 4 Discussion

The menace of diabetes mellitus has grown exponentially over the past few decades, which is reflected by the yearly increase in the number of prediabetic and diabetic patients, as well as the huge health expenditure (over 900 billion dollars) associated with the management of the disease. In fact, a recent report has shown over 300% increase in diabetes related health expenditure within the last 15 years (International Diabetes Federation, 2021). Medicinal plants have a long history in the treatment of chronic disease including diabetes and its related complications, and their effectiveness has necessitated rigorous exploration of the extracts or bioactive components from these plants as possible antidiabetic remedy (Makinde et al., 2020; Ajiboye et al., 2023). *L. sylvestris* has been shown to exert pharmacological properties such as antioxidant, alpha glucosidase inhibition and insulin secretagogue properties, thus making it a notable plant to be explored for its potential antidiabetic properties in models of type 2 diabetes.

One of the major reasons for the use of medicinal plants in treatment of chronic diseases like diabetes is due to their ability to supress free radicals owing to their excellent antioxidant properties. A huge number of reports have established a direct correlation between the bioactivity of medicinal plants and their antioxidant ability, which is obviously associated with the presence of natural antioxidant compounds mainly polyphenols such as phenolics and flavonoids (Shao et al., 2022; Lei et al., 2023). Generally, LSB showed anti-radical activities on DPPH and ABTS+, with moderate metal reducing capabilities in the FRAP assay. The anti-radical activity of LSB could be attributed to the presence of flavonoids and phenolic contents in the extract, which is obviously reflected in the results of the total phenolic and flavonoid content of the extract. These results agree with a previous report on *Lepionurus sylvestris* Blume extract (Phoopha et al., 2023).

Out of the several clinical approaches used in the treatment of diabetes, the inhibition of carbohydrate hydrolysing enzymes is considered as a corner stone. The inhibition of alpha glucosidase and alpha amylase enzymes delays the liberation of glucose from a carbohydrate diet, thus reducing postprandial blood glucose level (Assefa et al., 2019; Makinde et al., 2019). Although α-glucosidase inhibitors have been in clinical use for several decades, there are only limited number of these inhibitors (acarbose, miglitol, and voglibose) that have been approved for the treatment of diabetes. In addition, these α-glucosidase inhibitors are often associated with several side effects including gastrointestinal anomalies (Makinde et al., 2019; Daou et al., 2022). In line with scores of report on the efficacy of medicinal plants as α-glucosidase inhibitors, the results obtained from this study showcases the inhibitory activity of LSB against α-glucosidase enzyme, with a better efficacy than acarbose (Makinde et al., 2019; Phoopha et al., 2023; Zhang et al., 2023).

Type 2 diabetes mellitus is a multiplex metabolic disorder arising from insulin resistance and malfunctioning of the beta cells of the pancreas, ultimately resulting in hyperglycemia, disordered fat and lipid metabolism (Makinde et al., 2020; Folorunso et al., 2022). Glucotoxicity, a hallmark of impaired β-cell function during hyperglycemia causes endoplasmic reticulum and mitochondrial stress, which can subsequently induces oxidative stress and inflammation leading to series of diabetic comorbidity (Robertson et al., 2023). Thus, achieving proper glycaemic control is crucial, and it is the basis for the clinical treatment of diabetic patients.

Insulin resistance and hyperinsulinemia precede the development of T2DM and hyperglycemia. Insulin resistance which precede the development of T2DM results in a rapid surge in insulin demand, thus triggering the pancreatic β-cells to adapt to this new demand by increasing the mass and function of the β-cell to release sufficient insulin, thus maintaining normal glycemia This compensatory mechanism results in hyperinsulinemia, thus initiating the development of metabolic disorders (Zhang et al., 2019; Rachdaoui, 2020). This cycle ultimately exhaust the pancreas, leading to β-cell failure and decompensation, resulting in the inability of the pancreas to produce/secrete sufficient amount of insulin needed, leading to hyperglycemia (Rachdaoui, 2020). In this study, diabetic animals showed significant increase in blood glucose level, with a marked decline in serum insulin levels and impaired glucose tolerance. In addition, the body weight of the diabetic rats was decreased compared to healthy animals. The increase in the blood glucose level and reduction in insulin levels might be attributed to the damage of the pancreatic β-cells by streptozotocin (Huang et al., 2022; Olofinsan et al., 2023). The structural and functional integrity of the islets of Langerhans has a direct impact on effective glucose homeostasis (Halban et al., 2014; Olofinsan et al., 2023). Furthermore, the reduction in body weight in diabetes has been associated to excess protein tissue destruction, dehydration and decreased insulin production (Owolabi et al., 2011; Makinde et al., 2020). The progressive emaciation and muscle wasting in diabetes has been associated with increased protein catabolism due to insulin deficiency. Insulin stimulates muscle protein synthesis and impaired insulin secretion results in reduced protein availability due to increased proteolysis (Charlton and Nair, 1998; Vats et al., 2004). The ability of LSB to significantly lower hyperglycemia, improve body weight gain and glucose tolerance may be linked to its insulin secretory ability as suggested by the increased insulin levels, as well as increased mass and number of β-cells in the pancreas of the treated animals. The overall improvement in these parameters suggests enhanced glucose utilization and insulin sensitivity in the LSB treated diabetic groups.

Insulin resistance has been prominently linked to metabolic syndrome, which includes a clusters of abnormality including obesity, hyperlipidemia, and cardiovascular diseases (Hauner, 2002; Zhao et al., 2023). Dyslipidemia, an imbalance in the lipids levels leading to hyperlipidemia is one of the major anomaly experienced by diabetic patients, and it has been shown to strongly correlate with the onset and progression of microvascular complications, especially cardiovascular diseases (Olatunji et al., 2017; Salau et al., 2023). In this study, the increased serum levels of low-density lipoprotein, triglyceride and cholesterol, as well as depleted levels of high-density lipoprotein was evidenced in the diabetic model group. Low density lipoprotein are very susceptible to oxidative modification and the likelihood of these lipoprotein to be modified in diabetic situation is high due to increase in the generation of reactive oxygen species and oxidative stress. Furthermore the high inclination of low density lipoproteins for glycoproteins in the artery walls leads to the formation of plagues in the inner linings of the arterial wall, thus increasing the risk of atherosclerosis, stroke and myocardial infarction (Piko et al., 2023). On the other hand, accumulating evidences have portrayed HDL as a glucoregulatory lipoprotein due to its effects on glucose metabolism, pancreatic insulin secretion enhancement, glucose uptake as well as insulin sensitivity (Siebel et al., 2015). HDL is also involved in the clearance of cholesterol from peripheral tissues via reverse cholesterol transport to the liver for either redistribution to other tissues or excretion. It can also act as an antioxidant and anti-inflammatory lipoprotein (Femlak et al., 2017; Piko et al., 2023). In this work, we found substantial increase in serum lipid parameters including cholesterol, triglyceride and low density lipoprotein cholesterol and a decrease in HDL level in the diabetic rats, indicating altered lipid metabolism. However treatment with LSB substantially reduced the serum lipid profiles, as well as increased HDL levels in the treated diabetic animals.

Hyperglycemia in consonance with oxidative stress has been shown to instigate several pathological changes in the body leading to micro and macrovascular comorbidities that damages different body organs notably the kidney, liver, heart, brain and the foot (Giacco and Brownlee, 2010; Olatunji et al., 2018). Diabetic nephropathy and metabolic dysfunction–associated steatotic liver disease (MASLD) are complications affecting the kidney and liver in diabetes. Earlier studies have shown that elevated concentrations of liver transaminases in the blood is indicative of hepatocellular injury, which is a prominent occurrence in diabetes. Moreover, high levels of creatine and blood urea nitrogen has been strongly correlated with diabetic nephropathy (Ekperikpe et al., 2019; Song et al., 2021; Olofinsan et al., 2023). As such, the increased level of transaminases and kidney function enzymes (creatinine and blood urea nitrogen) observed in the diabetic model rats may imply the existence of diabetic induced liver and kidney complications, which is similar to reported literatures in diabetic models (Olatunji et al., 2023; Salau et al., 2023). LSB supplementation remarkably diminished the serological levels of hepatic and renal function markers in the treated rats, suggesting LSB’s protective roles on diabetes-associated hepatorenal damages.

Hyperglycemia has been shown to explicitly triggers multiple pathways that stimulates oxidative stress in various tissues. The close triad relationship between hyperglycemia, oxidative stress and inflammation in several pathological and metabolic dysfunction of organs involved in glucose metabolism accounts for the pathogenesis of diabetic complications (Osukoya et al., 2022; Qi et al., 2024). Increased demand for the synthesis and secretion of insulin due to high glucose levels in diabetic situations exhaust the beta cells, leading to endoplasmic reticulum stress, increased ROS generation and oxidative stress (Eguchi et al., 2021). The auto-oxidation of glucose generates free radicals, especially superoxide radicals, which is converted to hydrogen peroxide by SOD, while the hydrogen peroxide is subsequently broken down into oxygen and water by the enzyme catalase (Banerjee et al., 2020). However, the pancreas is liable to oxidative attack due to their high turnout of ROS as well as their low antioxidant defense capability (Gurgul-Convey et al., 2016; Eguchi et al., 2021). Furthermore, the beta cell toxicity of streptozotocin is mediated by an upsurge in ROS and nitric oxide production leading to increased oxidative stress, which overwhelms the antioxidant defense mechanism of the pancreas, leading to beta cell apoptosis (Raza and John, 2012). Hence, our findings that diabetes resulted in increased pancreatic lipid peroxidation, depleted GSH levels as well as reduced CAT and SOD activities were in line with previous studies (Banerjee et al., 2020; Huang et al., 2022; Shao et al., 2022). The results of this study indicated that pancreatic levels of GSH, CAT and SOD were significantly improved, while the levels of MDA was decreased in LSB treated rats. These results corroborated with several previous studies (Adam et al., 2016; Khamchan et al., 2018). Taken together, our findings suggested that treatment with LSB rescued diabetes induced oxidative stress in the pancreas.

In like manner, several studies have extensively shown that inflammation plays a critical role in the onset and progression of diabetes and its associated complications. Low grade inflammation in the pancreatic tissues stimulates insulin resistance, hyperglycemia and immune responses, leading to proinflammatory cytokine surge (Pop-Busui et al., 2016; Rohm et al., 2022). In addition, hyperglycemia further increases oxido-inflammatory markers, thus creating a vicious cycle between inflammation and oxidative stress (Luc et al., 2019; Banerjee et al., 2020). These vicious interplay between hyperglycemia, inflammation and oxidative stress is considered as one of the major backbone for the development of diabetes induced multiorgan damage (Ni et al., 2019; Osukoya et al., 2022; Wen et al., 2022). In this study, the observed increase in proinflammatory cytokines in the pancreas of the diabetic animals agrees with the findings from earlier studies (Banerjee et al., 2020; Mamdouh Hashiesh et al., 2023). The increased pancreatic proinflammatory cytokines were significantly decreased in LSB treated animals.

A number of compounds tentatively identified in LSB has been previously reported to have promising antidiabetic activities (Adhikari, 2021; Chelliah et al., 2021). For instance, kaempferol exhibits multiple antidiabetic mechanisms, including enhancing insulin secretion, improving glucose uptake, and reducing oxidative stress (Yang et al., 2022). Similarly, the lignan glycosides episyringaresinol exerted antidiabetic effects by improving insulin sensitivity and reducing inflammation (Akinmoladun et al., 2020). Another important compound ferulic acid has demonstrated antidiabetic effects by improving insulin sensitivity and protecting against diabetic complications through its antioxidant properties (Sri Balasubashini et al., 2003; Ghosh et al., 2018; Li et al., 2022). Furthermore, the monoterpene myrcene has been studied for its role in improving glucose tolerance and insulin sensitivity, suggesting potential benefits in diabetes management (Yang and Liao, 2021). Coumarins such as scoparone possess insulin-sensitizing properties and have been investigated for their potential to alleviate insulin resistance (Chen et al., 1994; Wang et al., 2017). Quinic acid have exhibited potential antidiabetic effects by improving insulin sensitivity and reducing blood glucose levels (Arya et al., 2014; Benali et al., 2022). Moreover, quinic acid has also displayed potential interaction score with enzymes DPP-IV and α-amylase in the *in silico* molecular docking studies. This provides insights into the possible mechanisms involved in the antidiabetic properties of quinic acid. Cyanogenic glycosides like lotaustralin have shown hypoglycemic effects by stimulating insulin release and enhancing glucose utilization (Manimurugan et al., 2023). Lotaustralin has also shown significant interaction against α-glucosidase in the molecular docking approach. Similarly, trigonelline has demonstrated antidiabetic properties by improving insulin sensitivity and reducing blood glucose levels. It may also protect pancreatic β-cells from glucotoxicity (Zhou et al., 2012; Hamden et al., 2013). This compound’s capability of coping with diabetes through the inhibition of antidiabetic enzymes has been rectified in the *in silico* study. Overall, it seems that the presence of important compounds from diabetes persepectives in LSB can be the contributory factor to the promising antidiabetic potential of the plant.

## 5 Conclusion

Summing up, the observed fructose/streptozotocin-induced hyperglycemia, hyperlipidemia, metabolic derangement and pancreatic damage in rats correlates with increased levels of oxido-inflammatory biomarkers. Whereas, it can be deduced that LSB could reverse metabolic derangement, hyperglycemia, glucose intolerance, biochemical, oxidative and inflammatory imbalances in the treated animals by virtue of its multitudinous bioactive constituents. These findings concluded that *Lepionurus sylvestris* Blume extract could be a potential agent for the treatment of diabetes. Further studies on the molecular mechanism of LSB extract need to be explored.

## Data Availability

The raw data supporting the conclusions of this article will be made available by the authors, without undue reservation.
